# Molecular epidemiological analysis and risk factors for acquisition of carbapenemase-producing *Enterobacter cloacae* complex in a Japanese university hospital

**DOI:** 10.1186/s13756-019-0578-3

**Published:** 2019-07-24

**Authors:** Nobuyuki Tetsuka, Aki Hirabayashi, Akane Matsumoto, Keisuke Oka, Yuki Hara, Hiroshi Morioka, Mitsutaka Iguchi, Yuka Tomita, Masato Suzuki, Keigo Shibayama, Tetsuya Yagi

**Affiliations:** 1 Department of Infectious Diseases, Nagoya University Hospital, 65 Tsurumai, Nagoya, Aichi 466-0065 Japan; 2 Department of Infectious Diseases, Nagoya University Graduate School of Medicine, Nagoya, Aichi Japan; 3 Antimicrobial Resistance Research Center, National Institute of Infectious Diseases, Tokyo, Japan; 4 Japanese Red Cross Nagoya Daini Hospital, Nagoya, Aichi Japan; 5 Department of Bacteriology II, National Institute of Infectious Diseases, Tokyo, Japan

**Keywords:** Molecular epidemiology, Carbapenem-resistant *Enterobacteriaceae*, Carbapenemase-producing *Enterobacteriaceae*, *Enterobacter cloacae* complex, β-Lactamase, Carbapenemase, Repetitive extragenic palindromic polymerase chain reaction, Multilocus sequence typing, Whole-genome sequencing, Plasmid

## Abstract

**Background:**

To clarify the molecular epidemiology of carbapenem-resistant *Enterobacter cloacae* complex (CREC) and the risk factors for acquisition of carbapenemase-producing *E. cloacae* complex (CPEC).

**Methods:**

Using clinical CREC isolates detected in a Japanese university hospital over 4 years, carbapenemase production was screened with phenotypic methods. Carbapenemase genes were analysed by PCR and sequencing. Molecular epidemiological analyses were conducted with repetitive extragenic palindromic (REP)-PCR and multilocus sequence typing (MLST). CRECs were identified to the subspecies level by *hsp60* sequencing. Whole-genome sequencing of plasmids was conducted. A case-control study was performed to identify risk factors for acquisition of CPEC among patients with CREC.

**Results:**

Thirty-nine CRECs including 20 CPECs carrying *bla*_IMP-1_ were identified. Patients with CPEC had longer hospital stay before detection (26.5 days vs. 12 days, *p* = 0.008), a urinary catheter (odds ratio [OR], 5.36; 95% confidence interval [CI], 1.14–30.9; *p* = 0.023), or intubation (OR, 7.53; 95% CI, 1.47–53.8; *p* = 0.008) compared to patients without CPEC. Four genetically closely related CPEC clusters were observed, which showed that three of four CPEC clusters corresponded to *E. asburiae* (ST 53), *E. hormaechei* subsp. *steigerwaltii* (ST 113 and ST 1047) and *E. cloacae* subsp. *cloacae* (ST 513) by MLST and *hsp60* sequencing. Seven representative plasmids shared structures with class I integron containing *bla*_IMP-1_ and IncHI2A replicon type.

**Conclusions:**

A longer hospital stay, presence of a urinary catheter, and intubation are risk factors for CPEC acquisition. In addition to horizontal transmission of genetically indistinguishable CPECs, IncHI2A plasmid carrying *bla*_IMP-1_ appeared to be transferred among genetically different ECs.

## Background

The emergence of carbapenem-resistant *Enterobacteriaceae* (CRE), especially carbapenemase-producing *Enterobacteriaceae* (CPE), has been increasing worldwide and is a clinical and public health threat [1]. Since September 2014, CRE infectious diseases have been involved in Category V Infectious Diseases identified by the Act on Prevention of Infectious Diseases and Medical Care for Patients Suffering Infectious Diseases in Japan. The National Epidemiological Surveillance of Infectious Diseases and Japan Nosocomial Infections Surveillance (JANIS) by the Ministry of Health, Labor and Welfare of Japan have been implemented for CRE using the criteria of either meropenem-minimum inhibitory concentration (MIC) ≥2 μg/mL or cefmetazole-MIC ≥64 μg/mL, in addition to imipenem-MIC ≥2 μg/mL. The annual report from JANIS 2017 showed that the prevalence of CRE is relatively low (0.27%). About half of CRE involves *Enterobacter* species (mainly *Enterobacter cloacae* complex (EC)). IMP-type β-lactamase is the most common carbapenemase in Japan [2, 3]. The latest results of surveillance of Japanese CRE in 2017 revealed that 239 CPE strains (28%) were detected, and 227 CPE strains carried *bla*_IMP,_ of which EC was the most common (74 strains). The IMP genotype was assessed in some strains, and IMP-1 (44%) and IMP-6 (56%) were detected [4].

CRE has been analysed predominantly in *Klebsiella pneumoniae* (KP) because of the initial global spread of CRE of KPC-type β-lactamase-producing KP strains. Most CRE research has focused on frequently detected species such as KP and *Escherichia coli* [5]. Among CRE, CPE bacteremia has a four-fold higher mortality rate within 14 days comparing to non-CPE CRE bacteremia [6]. It has been suggested that CPE is more likely to spread than non-CPE, and it is listed as one of the important research themes for infection control [7]. Recent meta-analyses of the clinical epidemiology of CRE showed that CREC is the second most common species among the studies that focused on a single species of the *Enterobacteriaceae* family [8]. In Japan as well, CREC is the second major species of CRE, and molecular characterization of CREC clinical isolates has been reported [9–11]. Further understanding of the molecular epidemiology of CREC and investigation of carbapenemase gene-carrying plasmids, which is the most important resistance mechanism of transmission, are needed to prevent the spread of CREC.

This study aimed to clarify the molecular epidemiology of CPEC isolates and their plasmids carrying carbapenemase genes detected in Nagoya University Hospital (NUH) and to analyse the risk factors for CPEC acquisition compared with CREC without carbapenemase production.

## Methods

### Study design and population

This was a single-centre, retrospective, observational study of hospitalized patients with positive cultures of CREC from April 1, 2012 to March 31, 2016 at NUH, a 1,035-bed tertiary acute care hospital in Japan. The first CREC isolate from a patient at NUH during the study period that met the CRE surveillance definition in Japan was included [3]. A case-control study was conducted to identify risk factors for acquisition of CPEC between patients who acquired CPEC and those who acquired non-CPEC.

### Data collection

Patient information was retrieved from patient electronic medical records. The parameters included demographics, background conditions and comorbidities, recent health care-associated exposure (such as stays in health care facilities), invasive procedures, the presence of indwelling devices, exposure to antimicrobials within 3 months prior to isolation of CREC, and the clinical outcome. Infectious clinical diagnosis was determined according to the information present in medical charts recorded by the attending doctor. The patients were determined to be colonisers if they did not have any signs or symptoms of infection based on information in their medical charts.

### Microbiological methods

Primary identification of bacterial species and antimicrobial susceptibilities was performed using an automated identification and susceptibility testing system (MicroScan WalkAway; Beckman Coulter, Brea, CA, USA) according to Clinical and Laboratory Standards Institute guidelines (document M100-S22), with dry plates (Beckman Coulter) in each case. EC isolates that showed either meropenem-MIC ≥2 μg/mL or cefmetazole-MIC ≥64 μg/mL, in addition to imipenem-MIC ≥2 μg/mL, according to the definition by the Act on Prevention of Infectious Diseases and Medical Care for Patients Suffering Infectious Diseases for CRE in Japan, were included [3].

### Screening of carbapenemase and sequencing of the carbapenemase gene

CRECs were screened to detect carbapenemase production using disc synergy tests, specific inhibitors, and the modified carbapenemase inactivation method (mCIM) [12]. All mCIM-positive CRECs were screened for the presence of *bla*_IMP-1_, *bla*_IMP-2_, *bla*_NDM-1_, *bla*_VIM-2_, and *bla*_KPC_ genes using PCR with primers as previously described [13–15]. When an isolate tested positive for the carbapenemase gene, its PCR product was sequenced at a commercial laboratory (Eurofin Genomics, Tokyo, Japan) and assembled with Sequencher DNA sequence analysis software (Gene Codes, Ann Arbor, MI, USA). Using its consensus sequence, the type of IMP was determined with BLAST (https://blast.ncbi.nlm.nih.gov/Blast.cgi).

### Repetitive extragenic palindromic polymerase chain reaction (REP-PCR)

DNA was isolated with an UltraClean Microbial DNA isolation kit (MoBio, San Diego, CA, USA) and used for experiments with the *Enterobacter* spp. fingerprinting kit (bioMerieux Japan, Tokyo, Japan) per the manufacturer’s procedure. PCR products were separated by electrophoresis using microfluidic lab-on-a-chip (Agilent Bioanalyzer 2100; Agilent, Santa Clara, CA, USA). Results were analysed using DiversiLab (bioMerieux Japan) on-line software employing the Pearson correlation method, which places more emphasis on the presence or absence of bands than on their intensity. ECs with a similarity of fingerprinting less than 95% were considered genetically different, and isolates with a similarity of > 98% were considered indistinguishable [16]. Isolates with a similarity between these values were judged manually using the pattern overlay option in the software. Isolates that were indistinguishable by DiversiLab testing belonged to clusters, and horizontal transmission was defined when an indistinguishable CREC was detected in different patients.

### *hsp60* PCR and sequencing

Amplification of *hsp60* was accomplished using previously described primers and conditions [17] with DNA extracted with the Cica Geneus DNA Extraction Reagent (Kanto Chemical, Tokyo, Japan). DNA sequencing was performed at a commercial laboratory (Eurofin Genomics) and assembled with Sequencher DNA sequence analysis software (Gene Codes). Based on the neighbour-joining tree of the *hsp60* sequences, detailed bacterial names were determined from 12 genetic clusters and an unstable sequence crowd using ClustalW (http://clustalw.ddbj.nig.ac.jp/). Reference strains and type strains were used for neighbour-joining tree of the *hsp60* sequences, and the genotypes were determined according to Hoffmann et al [17]. To identify members of *E. cloacae* complex to subspecies level, species and subspecies were referred to Chavda et al based on *hsp60* cluster result [18].

### Multilocus sequence typing (MLST)

DNA was isolated with Cica Geneus DNA Extraction Reagent (Kanto Chemical). Seven housekeeping genes were amplified using primer sets according to the method previously reported [18, 19]. DNA sequencing was performed at a commercial laboratory (Eurofin Genomics) and assembled with Sequencher DNA sequence analysis software (Gene Codes). Using its consensus sequence, the sequence type (ST) was determined with the *Enterobacter cloacae* locus/sequence definitions database (https://pubmlst.org/bigsdb?db=pubmlst_ecloacae_seqdef). If the sequence did not match the existing ST, new alleles and MLST profiles were registered in the *Enterobacter cloacae* locus/sequence definitions database (https://pubmlst.org/bigsdb?db=pubmlst_ecloacae_seqdef).

### Whole-genome sequencing

Plasmids carrying *bla*_IMP-1_ from seven representative isolates were subjected to whole-genome sequencing analysis on a MiniSeq system (Illumina, San Diego, CA, USA), and MinION nanopore sequencer (Oxford Nanopore Technologies, Oxford, UK) using the SQK-RBK004 kit and R9.4 flowcells to obtain complete sequences of plasmids carrying the *bla*_IMP-1_ gene. De novo assembly was performed with Unicycler [20] or Miniasm [21], error correction using Illumina reads with Unicycler or CLC Genomics Workbench v9.5.3 (QIAGEN, Hilden, Germany), and coding sequence (CDS) annotation with the PATRIC server (https://www.patricbrc.org). Linear comparison of *bla*_IMP-1_-carrying plasmid sequences was performed with BLAST and visualized with Easyfig (http://mjsull.github.io/Easyfig/). The *bla*_IMP-1_ gene, other antimicrobial resistance genes, type IV secretion system-associated genes for conjugation detected by the T346Hunter server [22], and mobile gene elements detected from CDS annotations were indicated.

### Statistical analysis

All analyses were performed using EZR [23]. The association of categorical variables with CREC patients was performed using Fisher’s exact test. For continuous data, Mann-Whitney tests were applied appropriately. Statistical significance was considered when the *p*-value was less than 0.05.

## Results

**Fig. 1:**
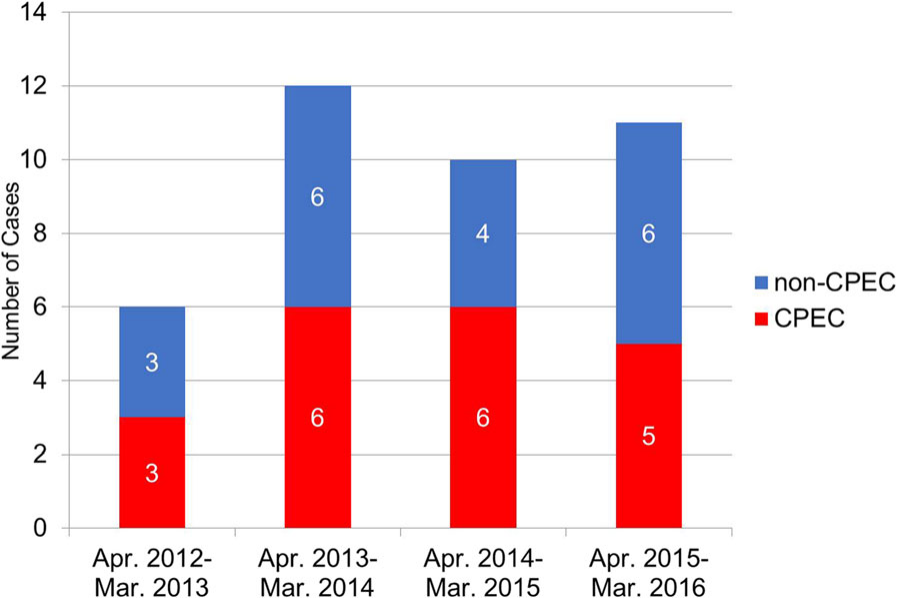
Annual incidence of carbapenem-resistant *Enterobacter cloacae* complex cases from April 2012 to March 2016 in Nagoya University Hospital. Abbreviations: CPEC, carbapenemase-producing *Enterobacter cloacae* complex

A total of 39 patients with non-duplicate CREC isolates were identified during the study period. Twenty CPECs among 39 CRECs were revealed with mCIM, and *bla*_IMP-1_ was identified in all CPEC isolates with negative PCR results for *bla*_IMP-2_, *bla*_NDM-1_, *bla*_VIM-2_, and *bla*_KPC._ The annual incidence of the CPEC cases among CREC cases is shown in Fig. 1. The number of CREC cases was six in the first year and ten to eleven cases per year in the subsequent 3 years. About half of CREC was CPEC every year during the study period.

Baseline characteristics and prior health care exposure of the patients with CPEC and non-CPEC are shown in Table 1. CREC isolates were detected most commonly in patients aged 65–79 years old. Almost all individuals had at least one underlying comorbid condition, and approximately 75% had a history of surgery. Patients with CPEC were more likely to have had a longer hospital stay (26.5 days vs. 12 days, *p* = 0.008), to have had a urinary catheter (odds ratio [OR], 5.36; 95% confidence interval [CI], 1.14–30.9; *p* = 0.023), and to have been intubated (OR, 7.53; 95% CI, 1.47–53.8; *p* = 0.008) before a positive culture result.

**Table 1:** Baseline characteristics and health care exposure of carbapenem-resistant *Enterobacter cloacae* complex cases

	CREC (*n* = 39)				OR (95% CI)	*p*
	CPEC (*n* = 20)		Non-CPEC (*n* = 19)			
Baseline characteristics						
Female sex	8		7		1.14 (0.26–5.05)	1
Age (years), median (range)	65	(0–82)	71	(0–89)		0.112
Age group (years)						0.431
0–18	3		1			
19–49	3		1			
50–64	4		2			
65–79	8		13			
≧80	1		2			
Days from admission to positive culture (days), median (range)	26.5	(1–352)	12	(0–52)		0.008
Underlying conditions	20		17			0.231
History of surgery	17		12		3.20 (0.58–23.2)	0.155
Any malignancy	6		9		0.49 (0.10–2.12)	0.333
Chronic renal insufficiency	4		4		0.93 (0.15–6.04)	0.939
Neurological disorder	5		3		1.75 (0.28–13.3)	0.695
Congestive heart failure	3		4		0.67 (0.08–4.67)	0.695
Diabetes	3		3		0.94 (0.11–8.11)	1
Transplant recipient	3		1		3.09 (0.22–176)	0.605
Cirrhosis	1		1		0.95 (0.01–78.4)	1
Decubitus or pressure ulcer	0		1			0.487
Chronic lung disease	1		0			1
Liver failure	1		0			1
Urinary tract problems or abnormalities	0		1			0.487
Myocardial infarction	1		0			1
HIV positive	0		1			0.487
Health care exposure						
Administration of antibiotics within 30 days	18		16		1.67 (0.17–22.3)	0.661
Acute care hospitalization within 3 months	10		13		0.47 (0.10–2.03)	0.333
Indwelling devices (2 calendar days prior to culture)	18		12		5.03 (0.78–57.7)	0.065
Central venous catheter	12		7		2.50 (0.60–11.4)	0.205
Nasogastric tube	11		7		2.06 (0.49–9.17)	0.341
Drainage tube	9		8		1.12 (0.27–4.80)	1
Intraperitoneal drainage tube	6		6		0.93 (0.19–4.50)	1
Chest drainage tube	4		1		4.34 (0.38–233)	0.342
Percutaneous transhepatic biliary drainage tube	3		1		3.09 (0.22–176)	0.605
Endoscopic nasobiliary drainage tube	1		2		0.46 (0.007–9.51)	0.605
Urinary catheter	12		4		5.36 (1.14–30.9)	0.023
Intubation (include tracheostomy)	12		3		7.53 (1.47–53.8)	0.008
Haemodialysis	2		4		0.43 (0.03–3.46)	0.407

Note. *OR* Odds ratio, *CI* Confidence interval, *CREC* Carbapenem-resistant *Enterobacter cloacae* complex, *CPEC* Carbapenemase-producing *Enterobacter cloacae* complex

The distributions of the culture source, infection types, and outcome of CREC cases are shown in Table 2. CRECs were commonly isolated from non-sterile samples (34/39; 87.2%), and CPECs were detected more frequently from sputum than non-CPECs (OR, 4.40; 95% CI, 0.94–25.0; *p* = 0.048). About a half of CREC cases, including five cases (5/39; 12.8%) detected from sterile sites, were considered the cause of infection (21/39; 53.8%), and pneumonia (7/39; 17.9%) and peritonitis (6/39; 15.4%) were the most common.

**Table 2:** Culture source, clinical diagnosis, and outcome among carbapenem-resistant *Enterobacter cloacae* complex cases

	CREC (*n* = 39)		OR (95% CI)	*p*
	CPEC (*n* = 20)	Non-CPEC (*n* = 19)		
Culture source				
Sterile site	2	3		
Blood	1	2	0.46 (0.007–9.51)	0.605
Abscess	1	0		1
Vascular graft	0	1		0.487
Non-sterile site	18	16		
Sputum	11	4	4.40 (0.94–25.0)	0.048
Peritoneal fluid (not punctured)	2	4	0.43 (0.03–3.46)	0.407
Urine	1	3	0.29 (0.05–4.02)	0.342
Bile	1	2	0.46 (0.007–9.51)	0.605
Oral swab	0	1		0.487
Stool	2	3	0.60 (0.04–5.96)	0.661
Clinical diagnosis				
Colonisation	11	7	2.06 (0.49–9.17)	0.341
Infection	9	12		
Pneumonia	5	2	2.76 (0.38–33.1)	0.407
Peritonitis	1	5^a^	0.15 (0.003–1.60)	0.092
Pyelonephritis	0	3		0.106
Bacteremia	1	2^a^	0.46 (0.007–9.51)	0.605
Cholangitis	1	0		1
Vascular graft infection	0	1		0.487
Abscess	1	0		1
Outcome				
Required intensive care unit stay in the 7 days after positive culture	10	7	1.67 (0.40–7.48)	0.523
Died at the end of the 30-day evaluation	1	2	0.46 (0.007–9.51)	0.605
Among sterile site positive culture	0	1		
Among non-sterile site positive culture	1	1		
Died during hospitalisation	3	2	1.48 (0.15–19.9)	1
Among sterile site positive culture	1	1		
Among non-sterile site positive culture	2	1		

^a^One case had peritonitis with bacteremia

Note. *OR* Odds ratio, *CI* Confidence interval, *CREC* Carbapenem-resistant *Enterobacter cloacae* complex, *CPEC* Carbapenemase-producing *Enterobacter cloacae* complex

About half of CREC patients (17/39; 43.6%) required a stay in the intensive care unit within 7 days after CREC was cultured, but no significant difference was found between CPEC and non-CPEC cases. Regardless of the culture source, the mortality rate within 30 days or during hospitalization did not differ significantly between CPEC cases and non-CPEC cases.

**Fig. 2:**
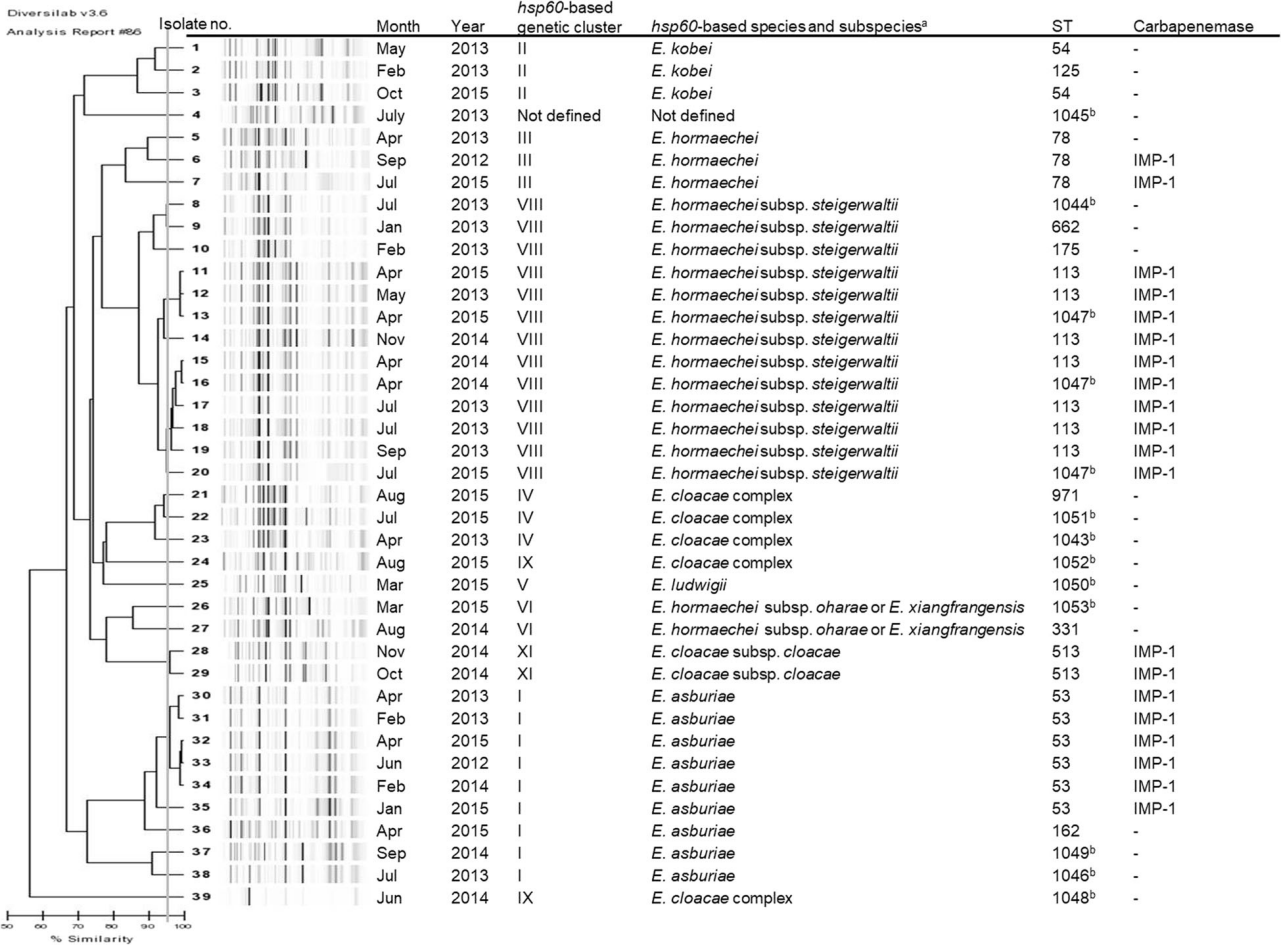
Phylogenetic tree based on repetitive extragenic palindromic (REP)-PCR of carbapenem-resistant *Enterobacter cloacae* complex with *hsp60*-based genetic cluster, subspecies, sequence type (ST) classified by multilocus sequence typing and identification of the carbapenemase gene. Genetic relatedness was determined by a similarity cut-off > 95%. ^a^Species and subspecies were determined with reference to the report by Chavda et al. ^b^Newly registered in this study

A molecular epidemiological study with REP-PCR, *hsp60*, and MLST was performed on all CREC isolates (Fig. 2). REP-PCR differentiated 27 unique genotypes including four CPEC clusters (isolates No. 11–13, No. 15–19, No. 28–29, and No. 30–34) with a similarity cut-off > 95%. Three of four genetically similar CPEC clusters consisted of isolates detected in different years or from patients without an apparent epidemiological relationship. *hsp60* sequencing analysis revealed 10 of the 12 genotypes described so far.

Isolates belonging to cluster VIII (=*E. hormaechei* subsp. *steigerwaltii*) were the most common, accounting for 33.3% [13] of all isolates, followed by those belonging to cluster I (= *E. asburiae*) (9/39; 23.1%). Only one isolate (2.6%) was not classified in Hoffmann cluster. Cluster VII (= *E. hormaechei* subsp. *hormaechei*), cluster X (=*E. nimipressuralis*) and cluster XII (=*E. cloacae* subsp. *dissolvens*) were not identified in the isolates of this study.

On the other hand, MLST analysis identified 31 unique STs, including 11 novel STs (ST 1043 to ST 1053). Isolates belonging to ST 113 were the most common, accounting for 17.9% (seven) of all isolates, followed by ST 53 (6/39, 15.4%). ST 113 and ST 1047 were different in only one of seven loci with seven single nucleotide polymorphisms. Other STs were different in at least three of seven loci.

Both MLST and *hsp60* sequencing resulted in a similar clustering pattern as REP-PCR and showed four CPEC clusters that corresponded to cluster I (= *E. asburiae*, ST 53), cluster VIII (=*E. hormaechei* subsp. *steigerwaltii*, ST 113 and ST 1047) and cluster XI (= *E. cloacae* subsp. *cloacae*, ST 513).

With reference to the phylogenetic tree, whole-genome sequencing was performed with seven representative strains (isolates No. 6, 11, 20, 28, 30, 32, and 35), and analysis of their plasmids was carried out (Table 3).

**Table 3:** Replicon type, integron type, antimicrobial resistance genes, heavy metal resistance genes and toxin/antitoxin system on the analysed plasmids

Plasmid	Host	Plasmid replicon	Integron type	Antimicrobial resistance genes	Heavy metal resistance genes			Toxin/antitoxin system
					Mercury	Arsenic	Tellurite	
pNUH12_ECL006_1	6	IncHI2A	Class 1	*aac(6′)-llc, bla*_IMP-1_*, qnrB6, sul1, tet(B)*	*+*	*+*	*+*	HipBA
pNUH15_ECL011_1	11	IncHI2A	Class 1	*aac(6′)-llc, bla*_IMP-1_*, qnrB6, sul1, tet(B)*	*+*	*+*	*+*	*–*
pNUH15_ECL020_1	20	IncHI2A	Class 1	*aac(6′)-llc, bla*_IMP-1_*, qnrB6, sul1, tet(B)*	*+*	*+*	*+*	HipBA
pNUH14_ECL028_1	28	IncHI2A	Class 1	*aac(6′)-la, bla*_IMP-1_*, sul1*	*+*	*+*	*+*	HipBA
pNUH13_ECL030_1	30	IncHI2A	Class 1	*aac(6′)-llc, bla*_IMP-1_*, qnrB6, sul1, tet(B)*	*+*	*+*	*+*	HipBA
pNUH15_ECL032_1	32	IncHI2A	Class 1	*aac(6′)-llc, bla*_IMP-1_*, sul1, tet(B)*	*+*	*+*	*+*	HipBA
pNUH15_ECL035_1	35	IncHI2A	Class 1	*aac(6′)-llc, bla*_IMP-1_*, qnrB6, sul1, tet(B)*	*+*	*+*	*+*	HipBA

**Fig. 3:**
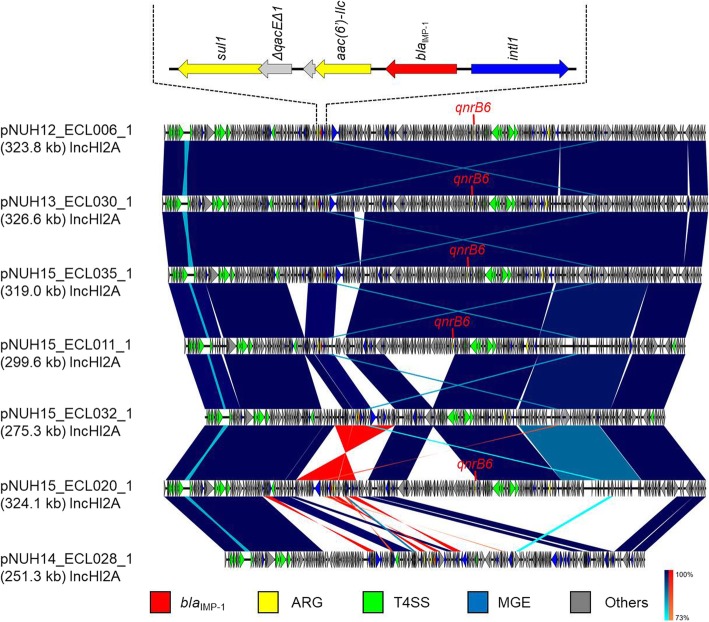
Linear comparison of *bla*_IMP-1_-carrying plasmid sequences from *Enterobacter cloacae* complex strains was performed with BLAST and visualized with Easyfig (http://mjsull.github.io/Easyfig/). Red, yellow, green, and blue arrows indicate the *bla*_IMP-1_ gene, other antimicrobial resistant genes (ARG), type IV secretion system (T4SS)-associated genes for conjugation, and mobile gene elements (MGE) detected from CDS annotations, respectively. Structural features of a class 1 integron containing *bla*_IMP-1_ are shown, and the structures are almost identical in six plasmids, except for pNUH14_ECL028_1

Six plasmids except for pNUH14_ECL028_1 had almost identical conjugation elements, mobile elements, and a class 1 integron containing *bla*_IMP-1_, *aac(6′)-IIc*, and *sul1* (Fig. 3). pNUH14_ECL028_1 also had a class 1 integron containing *bla*_IMP-1_, *sul1*, and *aac (6′)-1a* instead of *aac (6′)-IIc*, and did not have identical conjugation elements and mobile elements compared with the other six plasmids. The insertion sequence IS*Kpn7* was located upstream of a class 1 integron, and the insertion sequence IS*1* was located downstream of the integron in five plasmids (pNUH12_ECL006_1, pNUH13_ECL030_1, pNUH15_ECL035_1, pNUH15_ECL032_1 and pNUH15_ECL020_1). All seven plasmids carry heavy metal resistance genes for mercury, arsenic and tellurite. All plasmids except for pNUH15_ECL011_1 carry HipBA toxin/antitoxin system.

The data have been deposited with links to BioProject accession number PRJDB7521 in the DDBJ BioProject database. BioSample metadata are available in the DDBJ BioSample database under accession numbers SAMD00143514 to SAMD00143520. The sequence data of plasmids are available in the DDBJ/EMBL/GenBank databases under the accession numbers AP019382 to AP019388.

## Discussion

Molecular epidemiological analysis and genetic characterization of CREC and risk factors for CPEC acquisition over 4 years in a Japanese university-affiliated hospital were performed. Among the patients in whom CREC was detected, long-term hospitalization, the presence of an indwelling urinary catheter, and intubation were associated with isolation of CPEC. Comparison of the fingerprinting patterns by REP-PCR with MLST and *hsp*60-based genotyping showed general concordance except for isolate No. 39. REP-PCR experiments were conducted repeatedly, but the results were same. The reason why only this isolate did not show concordance is unclear. Molecular epidemiological analysis revealed that genetically indistinguishable CPECs were detected from patients without any epidemiological relationship who were hospitalized in different years, suggesting horizontal transmission, whereas non-CPECs were mostly genetically distinguishable. All CPECs carried *bla*_IMP-1_, and interestingly, even genetically distinct CPECs carried highly similar IncHI2A plasmids harboring *bla*_IMP-1_. These results suggest that the spread of CPECs in the hospital was due to complex mechanisms of a combination of clonal and plasmid transmission. Although we detected no single major clone like ST 235 in *K. pneumoniae*, among CRECs, CPECs were more likely to be clonally spread. As shown in this study, the clonal characteristics of patients who acquired CPEC compared with patients with non-CPEC, such as prolonged hospitalization or indwelling devices like a urinary tract catheter or intubation, may indicate a greater chance for acquiring of CPECs. Moreover, plasmid analysis of the representative CPEC isolates revealed common backbone structures, IncHI2A replicon type, class I integron containing *bla*_IMP-1_ and heavy metal resistance genes and toxin/antitoxin system. Intriguingly, this structure was highly similar to that of pMTY11043 IncHI2 detected from *Enterobacter hormaechei* (GenBank accession number AP081352.1) reported in Tokyo, Japan [9]. As reported in the study [9], the IncHI2A plasmids may be more likely to have common genes for toxin-antitoxin systems and heavy metal resistance, resulting in maintenance of the plasmids, especially in hospital environments [24, 25]. As few studies about CPECs have analysed their plasmids [9, 26, 27], further investigation of molecular epidemiology and plasmid distribution of CPECs at a regional or countrywide level is warranted.

The tendency for clonal transmission of CPECs presented in this study supported the validity of infection control policies focusing on CPE according to the CPE Toolkits published by the Centres for Disease Control and Prevention of England [28, 29]. In our hospital, according to the instructions from the Ministry of Health, Labor and Welfare in Japan, once a CPE not non-CPE CRE, was detected, active surveillance culture of the inpatients in the same ward as the index case was conducted for early detection of asymptomatic carriers. In addition, stringent contact precautions were implemented in both colonised and infected patients to prevent further transmission [30]. However the results of transmission of CPECs between patients hospitalized in different periods and without any apparent epidemiological linkage pose some challenges for infection control in our hospital. Hidden environmental sources such as sink drainage could exist [31, 32], although previous environmental cultures at the responsible intensive care unit and wards found no contamination (data not shown).

This study has some limitations. First, the study was performed at a single centre, and the results cannot be generalized to other institutions. Second, only the *E. cloacae* complex was targeted in this study among CRE. Whether this result can be applied to other *Enterobacteriaceae* is unknown.

## Conclusions

Molecular epidemiology and the genetic background of plasmids conferring carbapenem resistance showed horizontal transmission of some clones and plasmids with a common backbone of the IncHI2A replicon type and class I integron containing *bla*_IMP-1_. Risk factors for CPEC acquisition are a longer hospital stay and use of indwelling devices, especially intubation and a urinary catheter. Early detection of CPEC and strict infection control measures upon detection including active surveillance culture for asymptomatic carriers are necessary to minimize the transmission of CPECs.

## Data Availability

All the dataset of this article is available from the corresponding author if reasonably requested.
